# Stability is essential for insecticidal activity of Vip3Aa toxin against *Spodoptera exigua*

**DOI:** 10.1186/s13568-022-01430-w

**Published:** 2022-07-14

**Authors:** Bai-Wen Fu, Lian Xu, Mei-Xia Zheng, Qing-Xi Chen, Yan Shi, Yu-Jing Zhu

**Affiliations:** 1 Agricultural Bio-Resources Research Institute, Fujian Academy of Agricultural Sciences, Fuzhou, 350003 China; 2 School of Life Sciences, Xiamen University, Xiamen, 361005 China

**Keywords:** Vip3A, Proteolysis, Molecular dynamics simulation, Insecticidal activity

## Abstract

Vegetative insecticidal proteins 3A (Vip3A) were important insecticidal proteins for control of lepidopteran pests. Previous study demonstrated that Vip3Aa and Vip3Ad showed significant difference in insecticidal activities against *Spodoptera exigua*, while the molecular mechanism remained ambiguous. Here we demonstrated that the difference in insecticidal activities between Vip3Aa and Vip3Ad might be caused by the difference in stability of Vip3Aa and Vip3Ad in *S. exigua* midgut protease. Vip3Aa was quite stable while Vip3Ad could be further degraded. Molecular dynamics simulation revealed that Vip3Aa was more stable than Vip3Ad, with smaller RMSD and RMSF value. Amino acid sequence alignment indicated that three were three extra prolines (P591, P605 and P779) located on Vip3Aa. We further identified that residue P591 played a crucial role on stability and insecticidal activity of Vip3Aa. Taken together, our study demonstrated that the stability was essential for the insecticidal activity of Vip3A toxins, which might provide new insight into the action mode of Vip3A toxins and contribute to the design Vip3A variants with improved stability and insecticidal activity.

**Supplementary Information:**

The online version contains supplementary material available at 10.1186/s13568-022-01430-w.

## Introduction

Vegetative insecticidal proteins 3A (Vip3A), secreted from *Bacillus thuringiensis* (Bt) during their vegetative growth stage, are important insecticidal proteins that show toxicity against a wide variety of lepidopteran pests (Chakrabarty et al. 2020; Chakroun et al. 2016a, b; Gupta et al. 2021). It was worth mentioning that Vip3A proteins displayed synergistic effect when coupling with Cry toxins for the control of agricultural pests (Wang et al. 2020; Lemes et al. 2014). It was also reported that Vip3A proteins were very active against insect species of *Spodoptera*, which showed low susceptibility to Cry proteins (Zhu et al. 2006; Baranek et al. 2015; Song et al. 2016). Furthermore, because Vip3 proteins did not share the binding sites with Cry toxins, it was not easy for target pests to develop cross-resistance between Vip3A and Cry toxins (Walsh et al. 2014; Jackson et al. 2007). These properties made Vip3A toxins suitable candidates to complement Cry toxins in Bt crops to broaden the insecticidal spectrum and for resistance management purposes.

Up to date, there are 10 Vip3A subtypes, including Vip3Aa to Vip3Aj, have been identified and reported (Crickmore et al. 2022). The different Vip3A proteins exhibited significant differences in toxicity and insecticidal spectrum against target insects (Boukedi et al. 2018; de Escudero et al. 2014; Hernández-Martínez et al. 2013). For example, Vip3Aa, Vip3Ae and Vip3Af were toxic to *Spodoptera littoralis*, with LC_50_ of 228.42 ng/cm^2^, 65.71 ng/cm^2^, and 388.90 ng/cm^2^, respectively. By contrast, Vip3Ad showed little insecticidal activity against this species (Boukedi et al. 2018). Vip3Aa exhibited toxicity against considerable number of lepidopteran species, while the insecticidal spectrum of Vip3Ab was relatively narrow (de Escudero et al. 2014; Hernández-Martínez et al. 2013). Similar to Cry toxins, Vip3A toxins were produced in protoxin form and could be cleaved into activated-toxin in target insect midgut (Sellami et al. 2015; Kunthic et al. 2017; Chakroun et al. 2016a, b). Some researches revealed that Vip3A activated-toxins were more susceptible to the action of proteases when compared with Cry toxins (Caccia et al. 2014; Abdelkefi-Mesrati et al. 2011). These findings suggested that excessive degradation might also affect the activities of Vip3A toxins. The binding ability of Vip3A toxin was also reported involving in the action of Vip3A toxins (Jiang et al. 2018; Liu et al. 2011). The analysis of different Vip3A toxins with different activity could contribute to better understanding of the insecticidal mechanism of Vip3A toxin and designing of Vip3A toxins with improved activity.

Previous study demonstrated that Vip3Aa and Vip3Ad showed significant difference in insecticidal activities against lepidopteran pest *Spodoptera exigua*, while the molecular mechanism was still unclear (Pan et al. 2017). Here we found that both of Vip3Aa and Vip3Ad could be cleaved into activated-toxins by *Spodoptera exigua* midgut protease and their cleavage sites were the same. However, Vip3Aa activated-toxin was stable when incubation with *S. exigua* midgut protease while Vip3Ad activated-toxin was less stable and could be further degraded. Molecular dynamics simulation also revealed that Vip3Aa was more stable than Vip3Ad. Amino acid sequence alignment displayed that three were three extra prolines (P591, P605 and P779) located on Vip3Aa and the mutation of residue P591 could lead to the decline of stability and insecticidal activity of Vip3Aa. Taken together, our study demonstrated that the stability of Vip3A toxin might play a key role for its insecticidal activity.

## Materials and methods

### Insects

A laboratory population of *S. exigua* larvae was purchased from Henan Jiyuan Baiyun Industry Co., Ltd, China. *S. exigua* larvae were fed with artificial diet provided by Hubei Biopesticide Engineering Research Center under the conditions of 26 ± 2 °C, 70% humidity and photoperiod of 14: 10 h (light: dark).

The *S. exigua* midgut proteases were prepared as previously described (Xu et al., 2016). 5 midguts were extracted from third instar larvae of *S. exigua* and followed by two washes with cold sodium chloride (128 mM). Midgut tissues were homogenized with 200 μL of phosphate-buffered saline (PBS) buffer (50 mM, pH 7.4) and centrifuged at 25,000 *g* for 30 min at 4 °C. The supernatants, which contained midgut proteases, were quantified by the BCA Protein Assay Kit (Beyotime, China) according to the instructions of the manufacturers.

### Expression and purification of Vip3Aa and Vip3Ad

*E. coli* BL21 (DE3) cells harboring pET30a-*vip3Aa* and pET30a*-vip3Ad* (the DNA sequences of *vip3Aa* and *vip3Ad* were listed in Additional file 1: Table S1) were grown in LB medium containing 35 μg/ mL kanamycin. The expression of Vip3Aa and Vip3Ad were induced overnight at 20 °C with 0.1 mM isopropyl-B-D-thiogalactopyranoside (IPTG) after OD_600 nm_ reached 0.4–0.6. Cells were pelleted at 6000 g at 4 °C and were resuspended with Tris–HCl buffer (100 mM Tris–HCl, 200 mM NaCl, 25 mM imidazole, pH 7.0). Cells were lysed by ultrasonication and the soluble protein was purified by a Ni-IDA Prepacked Column (Sangon, China) according to the manufacturer’s instructions. The purified Vip3Aa and Vip3Ad were exchanged into Sodium carbonate buffer (50 mM, pH 9.5) by a PD-10 desalination column (GE Healthcare, USA). Protein concentration was determined using a BCA Protein Assay Kit (Beyotime, China). Trypsin or *S. exigua* midgut juice was used to produce Vip3Aa and Vip3Ad activated-toxins as previous described (Pan et al. 2017).

### Bioassays

The insecticidal activities of Vip3Aa and Vip3Ad against first-instar larvae of *S. exigua* were estimated according to Pan et al. (2017). Six concentrations of toxins were set up, and sodium carbonate buffer (50 mM, pH 9.5) was served as a negative control. Twenty *S. exigua* larvae were treated with each concentration and the bioassays were repeated two times. Mortalities were recorded after 7 days. The LC_50_ values were analysed by SPSS 17.0 (Statistical Product and Service Solutions) using PROBIT analysis (Finney 1971).

### Transmission electron microscopy

Second-instar larvae of *S. exigua* were starved for 12 h and fed an artificial diet covered with Vip3Aa and Vip3Ad protoxins (4000 ng/cm^2^) for 48 h. After that, the midgut tissues of *S. exigua* were isolated, immediately fixed in 2.5% glutaraldehyde, and postfixed in 1% OsO_4_. The fixed midgut tissues were immerged into Epon for embedding. Ultrathin sections were sliced using a Leica ultramicrotome (EM UC7), stained with uranyl acetate and then lead citrate. The ultrastructure of the midgut epithelium was examined using a JEOL JEM-2100HC transmission electron microscope (Shao et al. 2016).

### Edman degradation sequencing analysis

N-terminal sequencing was conducted to determine the cleavage site of Vip3Ad processed by *S. exigua* midgut protease as previous described. Specially, 300 μg Vip3Ad activated-toxin was separated by SDS-PAGE electrophoresis, electro-transferred onto a PVDF membrane and submitted for amino acid sequencing using SHIMADZU automated protein/peptide sequencer (PPSQ-333A, JAPAN) (Xu et al. 2016).

### Proteolysis assay

Proteolysis assay was performed to evaluate the stability of Vip3Aa and Vip3Ad activated-toxins under *S. exigua* midgut juice. 20 μg Vip3Aa and Vip3Ad protoxin were mixed with *S. exigua* midgut juice (0.2 μg) in 50 μL final volume of sodium carbonate buffer (50 mM, pH 9.5). The mixtures were incubated at 30 °C for different incubation times, with constant shaking (about 40 rpm). The proteolytic reactions were stopped by boiling for 10 min and SDS-PAGE electrophoresis was used to assess the stability of Vip3Aa and Vip3Ad activated-toxins under *S. exigua* midgut juice.

### Site-directed mutagenesis

Site-directed mutagenesis was carried out to create Vip3Aa variants P591A, P605A and P779A by polymerase chain reaction (PCR) using PrimeSTAR Max DNA Polymerase (TaKaRa, Japan). Plasmid pET30a-*vip3Aa* was used as template. Primers used for mutagenesis were listed in Additional file 1: Table S2. The PCR program was as follows: 98 °C for 10 min for pre-denaturation, (98 °C for 15 s, 60 °C for 15 s, 72 °C for 2 min) × 25 cycles, and a final elongation step at 72 °C for 5 min. After reaction, PCR product (10 μL) were digested with the *Dpn* I (1U) at 37 °C for 1 h and further transformed into *E. coli* BL21 (DE3) competent to obtain the relevant mutants.

### Homology modeling and molecular dynamic simulation

The structure of Vip3Aa (PDB: 6TFJ) was obtained from PDB database (https://www.rcsb.org/). The homology model of and Vip3Ad was constructed by Swiss-Model (https://swissmodel.expasy.org/) with the crystal structure of Vip3Aa protoxin structure (PDB: 6TFJ) as the template (86.1% identity), and evaluated in SAVES (https://servicesn.mbi.ucla.edu/SAVES/).

The pretreatment of Vip3Aa and Vip3Ad for molecular dynamic (MD) were performed as previous study with AmberTools18. The system was heated from 0 to 300 K at constant volume in 50 ps with the protein restricted, equilibrated at constant pressure in 50 ps with the protein restricted and equilibrated for 500 ps without restriction of the protein. After equilibration, normal temperature and pressure (NPT) simulation was conducted for 20 ns to produce trajectories of MD simulation The root mean square deviation (RMSD) and the root mean square fluctuation (RMSF) were calculated for the protein backbone atoms using least-square fitting derived from the MD trajectories (Xu et al. 2020).

## Results

### Insecticidal activities of Vip3Aa and Vip3Ad against *S. exigua*

**Fig. 1:**
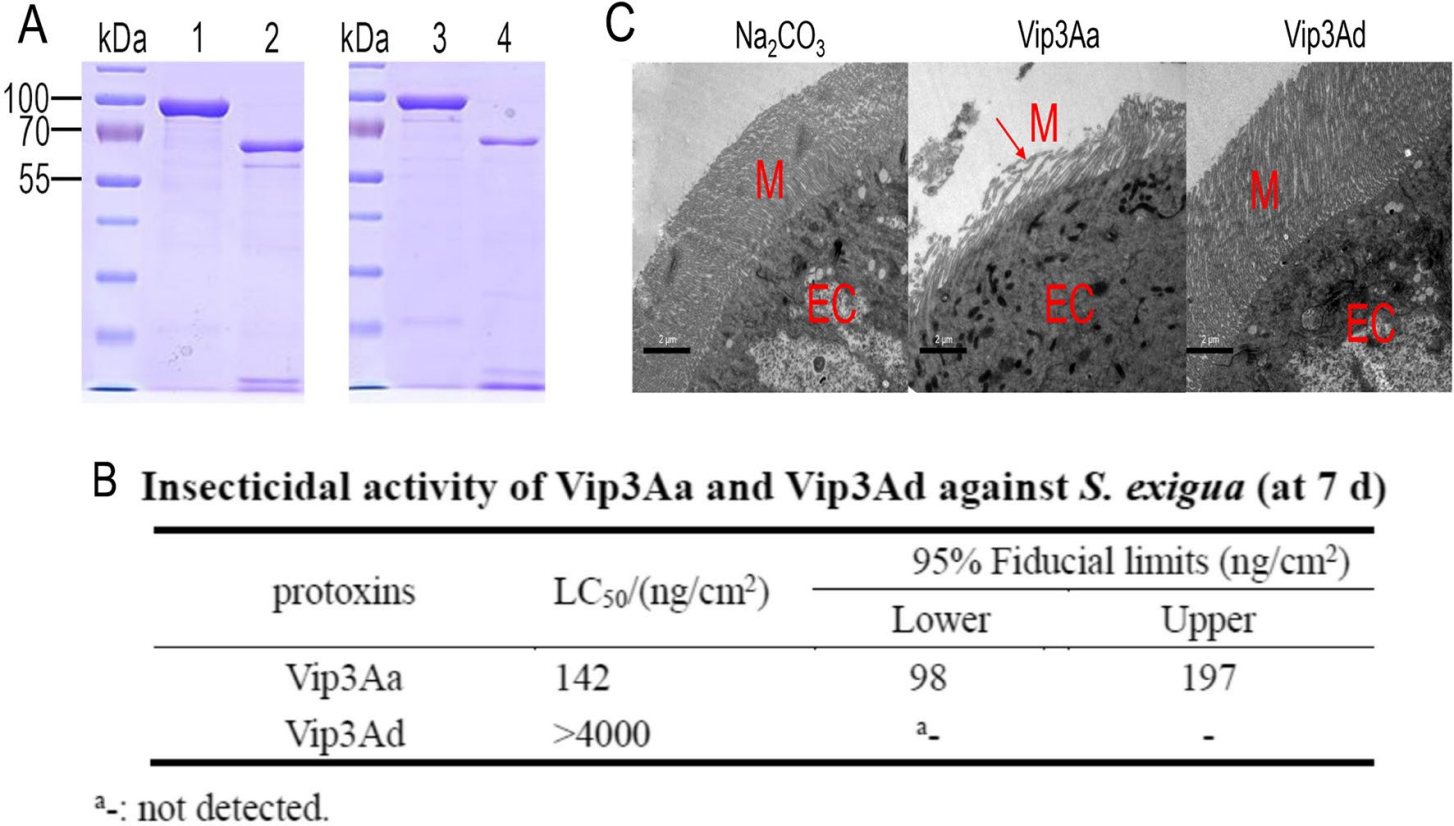
Studies on the activities of Vip3Aa and Vip3Ad against *S. exigua*. **A** Expression and purification of Vip3Aa and Vip3Ad. lane 1: Vip3Aa protoxin, lane 2: Vip3Aa activated-toxin, lane 3: Vip3Ad protoxin, lane 4: Vip3Ad activated-toxin. **B** Insecticidal activities of Vip3Aa and Vip3Ad against *S. exigua*. **C** The ultrastructure of *S. exigua* midgut epithelial cell treated with sodium carbonate buffer, Vip3Aa group and Vip3Ad group were observed using transmission electron microscope. EC: epithelial cell, M: microvilli

It was reported that Vip3Aa was toxic to *S. exigua* while Vip3Ad showed little insecticidal activity against *S. exigua*, although they shared more than 85% identity in amino acid sequence. To further investigate the molecular mechanism of the difference in insecticidal activities between Vip3Aa and Vip3Ad, we over-expressed Vip3Aa and Vip3Ad protoxin using *E. coli* Bl21 (DE3) expression system. SDS-PAGE indicated that Vip3Aa and Vip3Ad protoxins were both 89 kDa in molecular weight and could be cleaved into 65 kDa activated-toxins by *S. exigua* midgut juice (Fig. 1A). In fact, the 65 kDa activated-toxins were not a sole protein but composed of two attached fragments with molecular weight of 65 and 19 kDa, respectively (Jiang et al. 2020; Nuñez-Ramirez et al. 2020). We than evaluated the insecticidal activities of Vip3Aa and Vip3Ad against first instar larvae *S. exigua* larvae. As shown in Additional file 1: Fig S1, high concentration of Vip3Aa (400–1000 ng/cm^2^) could kill *S. exigua* larvae and low concentration of Vip3Aa (50–200 ng/cm^2^) could inhibit the growth of larvae. By contrast, it did not have any insecticidal or inhibitory activity under low concentration range of Vip3Ad (50–400 ng/cm^2^), inhibition effects were observed when the concentration of Vip3Ad increased to 1000 or 4000 ng/cm^2^ (Additional file 1: Fig S1). The LC_50_ value of Vip3Aa against *S. exigua* larvae was determined as 142 ng/cm^2^, much lower than that of Vip3Ad (> 4000 ng/cm^2^) (Fig. 1B). The disruptions of *S. exigua* midgut epithelium by Vip3Aa and Vip3Ad were also assessed by transmission electron microscopy. It demonstrated that serious pathological changes of the midgut epithelium and complete disintegration of the epithelial microvilli were clearly observed in Vip3Aa treatment group. While in Vip3Ad treatment group, the *S. exigua* midgut epithelial cells were integral, and the epithelial microvilli were neatly arranged, consistent with its nontoxicity against *S. exigua* larvae (Fig. 1C). The above results clearly confirmed the great distinction in insecticidal activities among Vip3Aa and Vip3Ad.

### Vip3Aa was more stable than Vip3Ad in *S. exigua* midgut juice

Vip3A toxins were produced in protoxin form and activated by insect midgut protease. Our previous study showed that the activation of Vip3Aa by *S. exigua* midgut protease was cleaved at K^198^, while the cleavage site of Vip3Ad was unknown (Zhang et al., 2018). In present study, Edman degradation sequencing was performed and showed that the N-terminal sequence of Vip3Ad activated-toxin was “D-X-P-P-A” (Additional file 1: Fig. S2), which matched the amino acid sequence of “^199^DSPPA^203^” in Vip3Ad. These results indicated that proteolysis of Vip3Aa and Vip3Ad were both cleaved at K^198^ and produced the relevant activated-toxins.

**Fig. 2:**
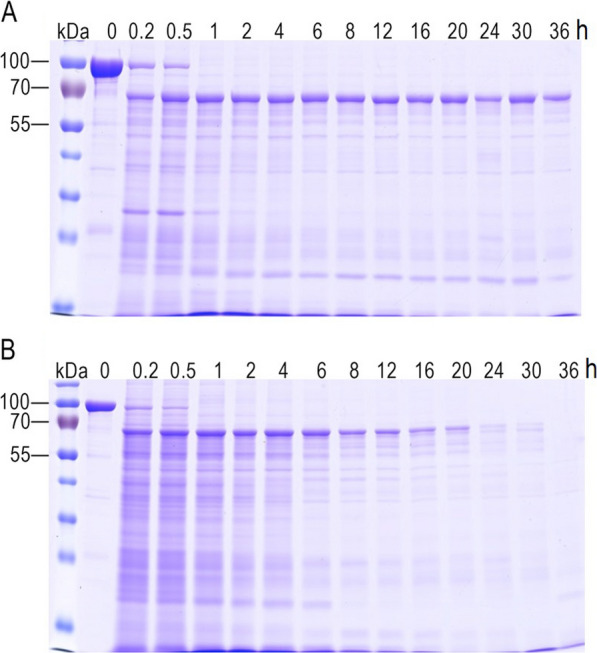
Time course of proteolytic processing of Vip3Aa (**A**) and Vip3Ad (**B**) by *S. exigua* midgut juice

For Cry toxins that also produced by *Bacillus thuringiensis*, it was reported that the stability of activated-toxin was associative with its insecticidal activity. We therefore estimated the time course degradation of Vip3Aa and Vip3Ad under 10% *S. exigua* midgut juice. As shown in Fig. 2A, Vip3Aa protoxin was cleaved into 70 kDa activated-toxin by *S. exigua* midgut protease and the activated-toxin was not further degraded, suggesting that it was stable when incubation with *S. exigua* midgut protease. For Vip3Ad protoxin, we also observed that the protoxin was gradually cleaved into 70 kDa activated-toxin. However, the Vip3Ad activated-toxin could be further degraded and completely disappeared when the incubation time was over 24 h (Fig. 2B). These results clearly demonstrated that Vip3Aa activated-toxin was more stable than Vip3Ad activated-toxin.

**Fig. 3:**
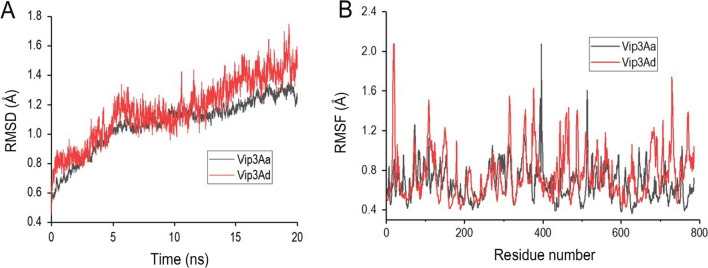
The RMSD (**A**) and RMSF (**B**) values of Vip3Aa and Vip3Ad based on 20 ns MD trajectories. The temperature was set as 303 K

To precisely interpret the differences in stability of Vip3Aa and Vip3Ad, these two toxins were subjected for 20 ns MD simulations at 303 K, respectively. The value of Root Mean Square Deviation (RMSD) and Root Mean Square Fluctuation (RMSF) were calculated. As shown in Fig. 3A, it demonstrated that both of Vip3Aa and Vip3Ad gradually became equilibrated at about 6 ns, however, the RMSD value of Vip3Ad fluctuated more than that of Vip3Aa, indicating that the overall structures of Vip3Aa was more stable than Vip3Ad. The RMSF results indicated that higher volatilities were clearly observed on residues 0–25, 370–382, 423–441 and 667–786 in Vip3Aa and Vip3Ad, also demonstrating that Vip3Aa was more stable than Vip3Ad (Fig. 3B).

### Residue P591 was essential for stability and insecticidal activity of Vip3Aa

To explore the mechanism for the different stability of Vip3Aa and Vip3Ad activated-toixn, we first compared the amino acid sequence of Vip3Aa and Vip3Ad toxin since they are about 15% differentiation in amino acid sequence. We found that three were three proline (P591, P605 and P779) located on Vip3Aa, while the corresponding residues on Vip3Ad were S, A and T, respectively (Additional file 1: Fig S3). As far as we know, proline had only one rotatable angle compared to the other 19 natural amino acids, resulting a relatively rigid structure and losing less entropy (Qu et al. 2022). This founding let us speculate that the more proline on Vip3Aa might contribute to the stability of Vip3Aa during the processing of midgut juice.

**Fig. 4:**
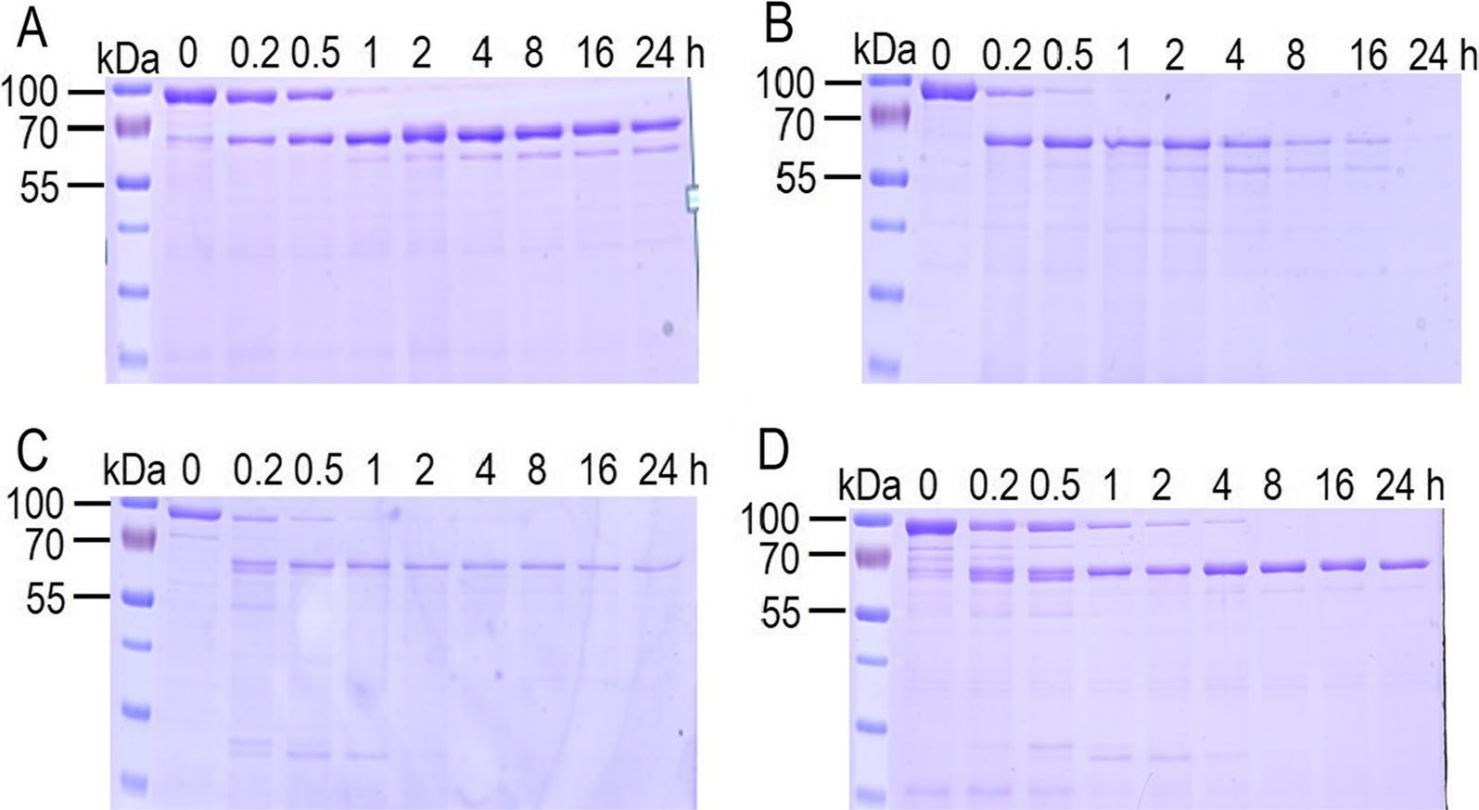
Time course of proteolytic processing of wild-type Vip3Aa (**A**), P591A (**B**), P605A (**C**) and P779A (**D**) by *S. exigua* midgut juice

To evaluate the residues P591, P605 and P779 on insecticidal activity of Vip3Aa, these three residues were mutated to alanine, constructing three mutants P591A, P605A and P779A. Time course degradation of wild-type and mutants Vip3Aa were performed. As shown in Fig. 4, wild-type and mutants Vip3Aa could be cleaved into 70 kDa activated-toxin. Observation after 24 h revealed that wild-type Vip3Aa, P605A and P779A activated-toxins were still existed and not further degraded, suggesting that they are stable under *S. exigua* midgut protease. By contrast, variant P591A was less stable in the *S. exigua* midgut protease and could be total degraded after 24 h. This result suggested that residue P591 was essential for the stability of Vip3Aa and its mutation could lead to more easily degradation of Vip3Aa when incubated with *S. exigua* midgut protease.

Bioassays were performed to further evaluate the insecticidal activities of wild-type, P591A, P605A and P779A Vip3Aa. As expected, the LC_50_ value of mutant P591A was 391 μg/cm^2^, higher when compared to wild-type, P605A, and P779A Vip3Aa, with LC_50_ values of 124, 195, and 135 ng/cm^2^, respectively (Table 1). These results indicated that residue P591A was essential for the stability and insecticidal activities of Vip3Aa.

**Table 1:** Insecticidal activity of wild-type and mutant Vip3Aa against *S. exigua*

Protoxins	LC_50_/(ng/cm^2^)	95% Fiducial limits (ng/cm^2^)	
		Lower	Upper
wild-type Vip3Aa	124	83	167
P591A	391	325	582
P605A	195	141	262
P779A	135	100	190

## Discussion

Previous study indicated that both Vip3Aa and Vip3Ad could bind to *S. exigua* BBMV, while the binding amount of Vip3Aa was more than Vip3Ad, which might be one reason for the difference in insecticidal activities between Vip3Aa and Vip3Ad (Pan et al. 2017). However, considering that Vip3Ad was nontoxic at all to *S. exigua*, we believed there were other factors resulting in the total inactivity of Vip3Ad.

The present study indicated that the cleavage site of Vip3Ad was at K^198^. This result demonstrated that the proteolysis activation of Vip3Aa and Vip3Ad were the same since previous study showed that the activation of Vip3Aa by *S. exigua* midgut protease was also cleaved at K^198^ (Zhang et al. 2018). The 65 kDa C-terminal fragments of Vip3A used to be considered as the toxic core. However, recent studies indicated that the N-terminal fragment (about 19 kDa) and the C-terminal fragment of Vip3A still bind together after digestion, and the N-terminus is required for the stability and toxicity of Vip3A (Jiang et al. 2020; Nuñez-Ramirez et al. 2020). We speculated that the stabilities of Vip3Aa and Vip3Ad were different and showed that Vip3Aa was more stable than Vip3Ad gut protease. Molecular dynamics simulation also revealed that Vip3Aa was more stable than Vip3Ad, with smaller RMSD and RMSF value. Interestingly, three were three extra prolines (P591, P605 and P779) located on Vip3Aa. To the best of our knowledge, the introduction of proline into proteins had been studied numerous times, generally with the aim of increasing stability (Qu et al. 2022). For example, the proline substitution in chitinase from *Paenibacillus pasadenensis* CS0611 resulted in a 26.3-fold increase in half-life at 50 °C and a 7.9 °C increase in half-inactivation temperature relative to wild-type enzyme (Xu et al. 2020). It also reported that the frequency of proline residues in thermophilic enzymes is higher than in mesophilic enzymes (Land et al. 2019). This founding let us speculate that the more proline on Vip3Aa might contribute to the stability of Vip3Aa during the processing of midgut juice. We further constructed three Vip3Aa mutants (P591A, P605A and P779A) and evaluated their stability and insecticidal activities compared with wild-type Vip3Aa. The result indicated that residue P591 played a crucial role on activity of Vip3Aa.

Taken together, our study demonstrated that the stability was essential for the insecticidal activity of Vip3A toxins, which might provide new insight into the action mode of Vip3A toxins and contribute to the design Vip3A variants with improved stability and insecticidal activity.

## Supplementary Information

**Additional file 1: Table S1.** Primer sequences used for the generation of Vip3Aa mutants. **Table S2.** DNA sequences of Vip3Aa and Vip3Ad. **Figure S1. **The insecticidal activities of Vip3Aa and Vip3Ad against first-instar larvae of *S. exigua*. **Figure S2. **N-terminal sequencing identified cleavage-sites of Vip3Ad processed by *Spodoptera exigua *midgut juice. (A) Spectrum of 19 PTH-amino acids standards; (B)-(F) N-terminal amino acid identification of Vip3Aa activated-toxin. **Figure S3. **Amino acid sequence alignment of Vip3Aa and Vip3Ad. The sequences in blue box represented the 65 kDa activated-toxins of Vip3Aa and Vip3Ad. The positions of three extra prolines (P591, P605 and P779) was indicated as (●).

## Data Availability

The additional files are available online.
