# Supplementary material for: Stability is essential for insecticidal activity of Vip3Aa toxin against Spodoptera exigua
Source: AMB Express. 2022 Jul 14;12:92. doi: 10.1186/s13568-022-01430-w (PMC9283630; doi:10.1186/s13568-022-01430-w)
Supplement: Supplementary file 1 — Additional file 1: Table S1. Primer sequences used for the generation of Vip3Aa mutants. Table S2. DNA sequences of Vip3Aa and Vip3Ad. Figure S1. The insecticidal activities of Vip3Aa and Vip3Ad against first-instar larvae of S. exigua. Figure S2. N-terminal sequencing identified cleavage-sites of Vip3Ad processed by Spodoptera exigua midgut juice. (A) Spectrum of 19 PTH-amino acids standards; (B)-(F) N-terminal amino acid identification of Vip3Aa activated-toxin. Figure S3. Amino acid sequence alignment of Vip3Aa and Vip3Ad. The sequences in blue box represented the 65 kDa activated-toxins of Vip3Aa and Vip3Ad. The positions of three extra prolines (P591, P605 and P779) was indicated as (●). [file 13568_2022_1430_MOESM1_ESM.docx]

**Stability was essential for insecticidal activity of Vip3Aa toxin against *Spodoptera exigua***

Bai-Wen Fu ^2^, Lian Xu ^1^, Mei-Xia Zheng ^1^, Qing-Xi Chen ^2^, Yan Shi ^2^*, and Yu-Jing Zhu ^1^*

^1^ Agricultural Bio-Resources Research Institute, Fujian Academy of Agricultural Sciences, Fuzhou 350003, China.

^2^ School of Life Sciences, Xiamen University, Xiamen 361005, China.

* Corresponding authors:

Tel/Fax: 0591-83723032. E-mail: [zyjingfz@163.com](mailto:zyjingfz@163.com) (Yu-Jing Zhu)

Tel/Fax: 0592-2184648. E-mail: [yshi@xmu.edu.cn](mailto:yshi@xmu.edu.cn) (Yan Shi)

**Table S1.** Primer sequences used for the generation of Vip3Aa mutants.

| primer | primer sequence (5’-3’) |
| --- | --- |
| P591A F | GTTAAAAgcaAAAACTGAGTATGTAATCCAA |
| P591A R | CAGTTTTtgcTTTTAACTTATCTCCAATAAA |
| P605A F | AGGAAAAgcaTCTATTCATTTAAAAGATGAA |
| P605A R | GAATAGAtgcTTTTCCTTTAACAGTATATTG |
| P779A F | TGGTGGTgcaATTGTACATTTTTACGATGTC |
| P779A R | GTACAATtgcACCACCATATAAATTATTCCC |

The mutant and replacement nucleotide sequences were shown in lower case text.

**Table S2.** DNA sequences of Vip3Aa and Vip3Ad.

| ***vip3Aa* DNA sequence, NCBI No. KR340473** |
| --- |
| ATGAACATGAATAATACTAAATTAAGCACAAGAGCCTTACCAAGTTTTATTGATTATTTTAATGGCATTTATGGATTTGCCACTGGTATCAAAGACATTATGAACATGATTTTTAAAACGGATACAGGTGGTGATCTAACCCTAGACGAAATTTTAAAGAATCAGCAGTTACTAAATGATATTTCTGGTAAATTGGATGGGGTGAATGGAAGCTTAAATGATCTTATCGCACAGGGAAACTTAAATACAGAATTATCTAAGGAAATATTAAAAATTGCAAATGAACAAAATCAAGTTTTAAATGATGTTAATAACAAACTCGATGCGATAAATACGATGCTTCGGGTATATCTACCTAAAATTACCTCTATGTTGAGTGATGTAATGAAACAAAATTATGCGCTAAGTCTGCAAATAGAATACTTAAGTAAACAATTGCAAGAGATTTCTGATAAGTTGGATATTATTAATGTAAATGTACTTATTAACTCTACACTTACTGAAATTACACCTGCGTATCAAAGGATTAAATATGTGAACGAAAAATTTGAGGAATTAACTTTTGCTACAGAAACTAGTTCAAAAGTAAAAAAGGATGGCTCTCCTGCAGATATTCTTGATGAGTTAACTGAGTTAACTGAACTAGCGAAAAGTGTAACAAAAAATGATGTGGATGGTTTTGAATTTTACCTTAATACATTCCACGATGTAATGGTAGGAAATAATTTATTCGGGCGTTCAGCTTTAAAAACTGCATCGGAATTAATTACTAAAGAAAATGTGAAAACAAGTGGCAGTGAGGTCGGAAATGTTTATAACTTCTTAATTGTATTAACAGCTCTGCAAGCAAAAGCTTTTCTTACTTTAACAACATGCCGAAAATTATTAGGCTTAGCAGATATTGATTATACTTCTATTATGAATGAACATTTAAATAAGGAAAAAGAGGAATTTAGAGTAAACATCCTCCCTACACTTTCTAATACTTTTTCTAATCCTAATTATGCAGAAGTTAAAGGAAGTGATGAAGATGCAAAGATGATTGTGGAAGCTAAACCAGGACATGCATTGATTGGGTTTGAAATTAGTAATGATTCAATTACAGTATTAAAAGTATATGAGGCTAAGCTAAAACAAAATTATCAAGTCGATAAGGATTCCTTATCGGAAGTTATTTATGGTGATATGGATAAATTATTGTGCCCAGATCAATCTGAACAAATCTATTATACAAATAACATAGTATTTCCAAATGAATATGTAATTACTAAAATTGATTTCACTAAAAAAATGAAAACTTTAAGATATGAGGTAACAGCGAATTTTTATGATTCTTCTACAGGAGAAATTGACTTAAATAAGAAAAAAGTAGAATCAAGTGAAGCGGAGTATAGAACGTTAAGTGCTAATGATGATGGGGTGTATATGCCGTTAGGTGTCATCAGTGAAACATTTTTGACTCCGATTAATGGGTTTGGCCTCCAAGCTGATGAAAATTCAAGATTAATTACTTTAACATGTAAATCATATTTAAGAGAACTACTGCTAGCAACAGACTTAAGCAATAAAGAAACTAAATTGATCGTCCCGCCAAGTGGTTTTATTAGCAATATTGTAGAGAACGGGTCCATAGAAGAGGACAATTTAGAGCCGTGGAAAGCAAATAATAAGAATGCGTATGTAGATCATACAGGCGGAGTGAATGGAACTAAAGCTTTATATGTTCATAAGGACGGAGGAATTTCACAATTTATTGGAGATAAGTTAAAACCGAAAACTGAGTATGTAATCCAATATACTGTTAAAGGAAAACCTTCTATTCATTTAAAAGATGAAAATACTGGATATATTCATTATGAAGATACAAATAATAATTTAGAAGATTATCAAACTATTAATAAACGTTTTACTACAGGAACTGATTTAAAGGGAGTGTATTTAATTTTAAAAAGTCAAAATGGAGATGAAGCTTGGGGAGATAACTTTATTATTTTGGAAATTAGTCCTTCTGAAAAGTTATTAAGTCCAGAATTAATTAATACAAATAATTGGACGAGTACGGGATCAACTAATATTAGCGGTAATACACTCACTCTTTATCAGGGAGGACGAGGGATTCTAAAACAAAACCTTCAATTAGATAGTTTTTCAACTTATAGAGTGTATTTTTCTGTGTCCGGAGATGCTAATGTAAGGATTAGAAATTCTAGGGAAGTGTTATTTGAAAAAAGATATATGAGCGGTGCTAAAGATGTTTCTGAAATGTTCACTACAAAATTTGAGAAAGATAACTTTTATATAGAGCTTTCTCAAGGGAATAATTTATATGGTGGTCCTATTGTACATTTTTACGATGTCTCTATTAAGTAA |
| ***vip3Ad* DNA sequence, NCBI No. KR263164** |
| ATGAACATGAATAATGCTAAATTAAATGCAAGGGCCTTACCAAGTTTTATTGATTATTTTAATGGTATTTATGGATTTGCCATTGGTATTAAAGACATTATGAATATGATTTTTAAAACGGATACAGGTAGTAATCTAACCCTAGACGAAATTTTAAAGAATCAGCAGTTACTAAATGAAATTTCTGGTAAATTGGATGGGGTAAATGGGAGCTTAAATGATCTTATCGCACAGGGAAACTTAAATACAGAATTAGCTAAGCAAATCTTAAAAGTTGCAAATGAACAAAATCAAGTTTTAAATGATGTTAATAACAAACTAGATACGATAAATTCGATGCTTAAAATATATCTGCCTAAAATTACATCTATGTTAAGTGATGTAATGAAACAAAATTATGTGCTAAGCTTACAAATAGAATACTTAAGTAAACAATTGCAAGAAATCTCCGACAAGCTAGATATTATTAACGTAAATGTGCTTATTAACTCTACGCTTACTGAAATTACACCTGCGTATCAACGAATGAAATATGTGAATGAAAAATTTGAAGAATTAACTTTTGCTACAGAAACCACTTTAAAAGTAAAAAAGGATAGCCCTCCTGCTGATATTCTTGACGAATTAACTGAATTAACTGAACTAGCGAAAAGTGTTACAAAAAATGACGTGGATGGTTTTGAATTTTACCTTAATACATTCCACGATGTAATGGTAGGAAATAATTTATTCGGTCGTTCAGCTTTAAAAACTGCTTCGGAATTAATTGCTAAAGAAAATGTGAAAACAAGTGGCAGTGAAGTAGGAAACGTTTATAATTTCTTAATTGTATTAACAGCTCTACAAGCAAAAGCTTTTCTTACTTTAACAACATGCCGAAAATTATTAGGCTTAGCGGATATTGATTATACTTCTATCATGAATGAGCATTTAAATAAGGAAAAAGAAGAATTTAGAGTAAACATCCTTCCCACACTTTCTAATACCTTTTCTAATCCTAATTATGCAAAAGCTAAGGGAAGTAATGAAGATACAAAGATGATTGTGGAAGCTAAACCAGGATATGTTTTGGTTGGATTTGAAATGAGCAATGATTCAATTACAGTATTAAAAGCATATCAAGCTAAGCTAAAAAAAGATTATCAAATTGATAAGGATTCGTTATCAGAAATAATATATAGTGATACGGATAAATTATTATGTCCGGATCAATCTGAACAAATATATTATACAAAGAACATAGCATTTCCAAATGAATATGTTATTACTAAAATTGCTTTTACAAAAAAAATGAACAGTTTAAGGTATGAGGCGACAGCGAATTTTTATGATTCTTCTACAGGGGATATTGATCTAAATAAGACAAAAGTAGAATCAAGTGAAGCGGAGTATAGTATGCTAAAAGCTAGTGATGATGAAGTTTACATGCCGCTAGGTCTTATCAGTGAAACATTTTTGAATCCAATTAATGGATTTAGGCTTGCAGTCGATGAAAATTCCAGACTAGTAACTTTAACATGTAGATCATATTTAAGAGAGACATTGTTAGCGACAGATTTAAATAATAAAGAAACTAAATTGATTGTCCCACCTAATGTTTTTATTAGCAATATTGTAGAGAATGGAAATATAGAAATGGACACCTTAGAACCATGGAAGGCAAATAATGAGAATGCGAATGTAGATTATTCAGGCGGAGTGAATGGAACTAGAGCCTTATATGTTCATAAGGATGGTGAATTCTCACATTTTATTGGAGACAAGTTGAAATCTAAAACAGAATACTTGATTCGATATATTGTAAAAGGAAAAGCTTCTATTTTTTTAAAAGATGAAAAAAATGAAAATTACATTTATGAGGATACAAATAATAATTTAGAAGATTATCAAACTATTACTAAACGTTTTACTACAGGAACTGATTCGACAGGAGTTTATTTAATTTTTAATAGTCAAAATGGAGATGAAGCTTGGGGAGATAACTTTATTATTTTGGAAATTAGTCCGTGTGAAAAGTTATTAAGTCCAGAATTAATTAAAACAGATAAATGGAATAGTACGGGATCAACTTATATTAGCGATGATAGACTCACTCTTTATCGGGGAGGACGAGGAATTTTAAAGCAAAACCTTCAATTAGACGGTTTTTCAACTTATAGAGTCAATTTTTCTGTGGACGGAGATGCTAATGTAAGGATTCGTAATTCTAGGGAAGTGTTACTTGAAAAAAGATATTTGAACCGTAAAGGTGTTTCTGAAATGTTTACTACAAAATTTGATAAAGATAACTTTTATGTAGAGCTTTCTCAAGGGGATAATCTTGGTACTATTGTACATTTTTATGATTTCTCTATTAAATAA |

**Fig S1**


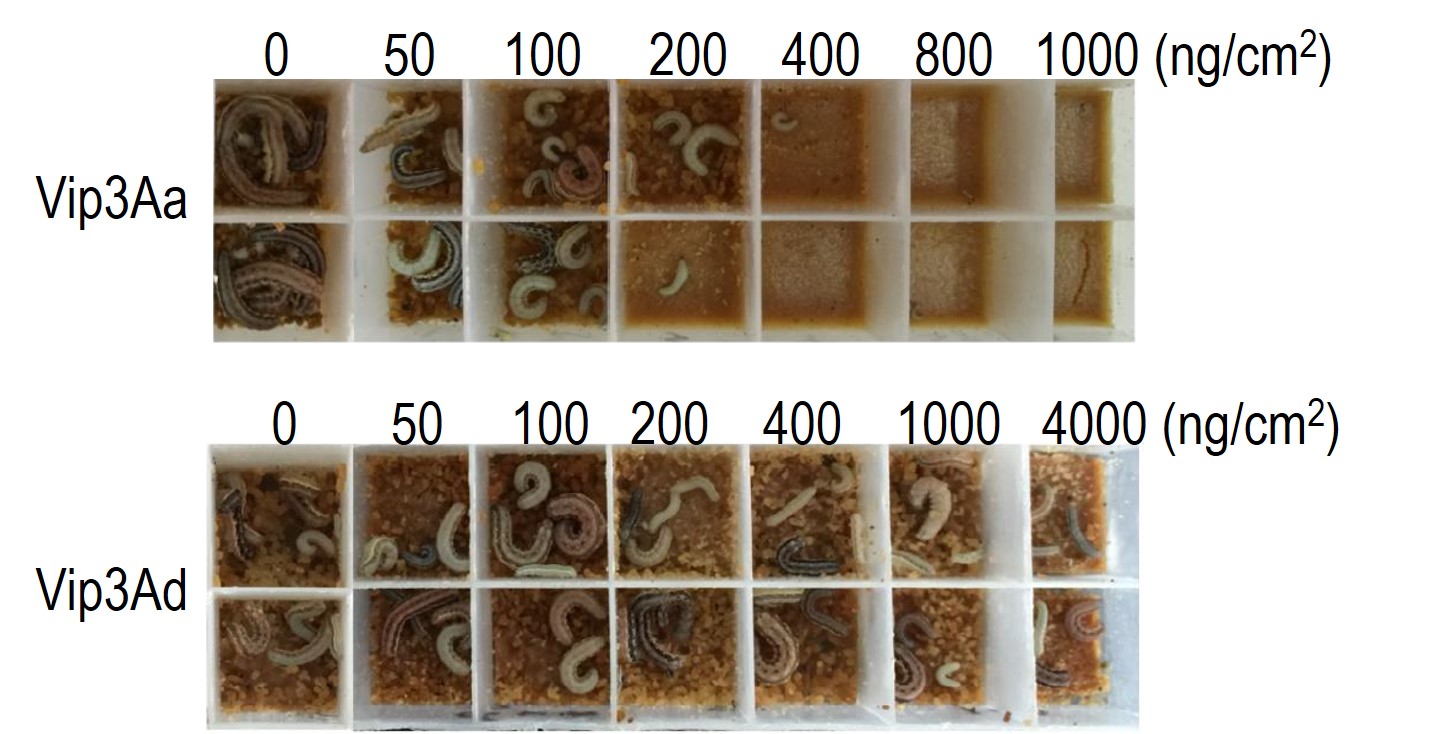


**Fig S1.** The insecticidal activities of Vip3Aa and Vip3Ad against first-instar larvae of *S. exigua*.

**Fig S2**


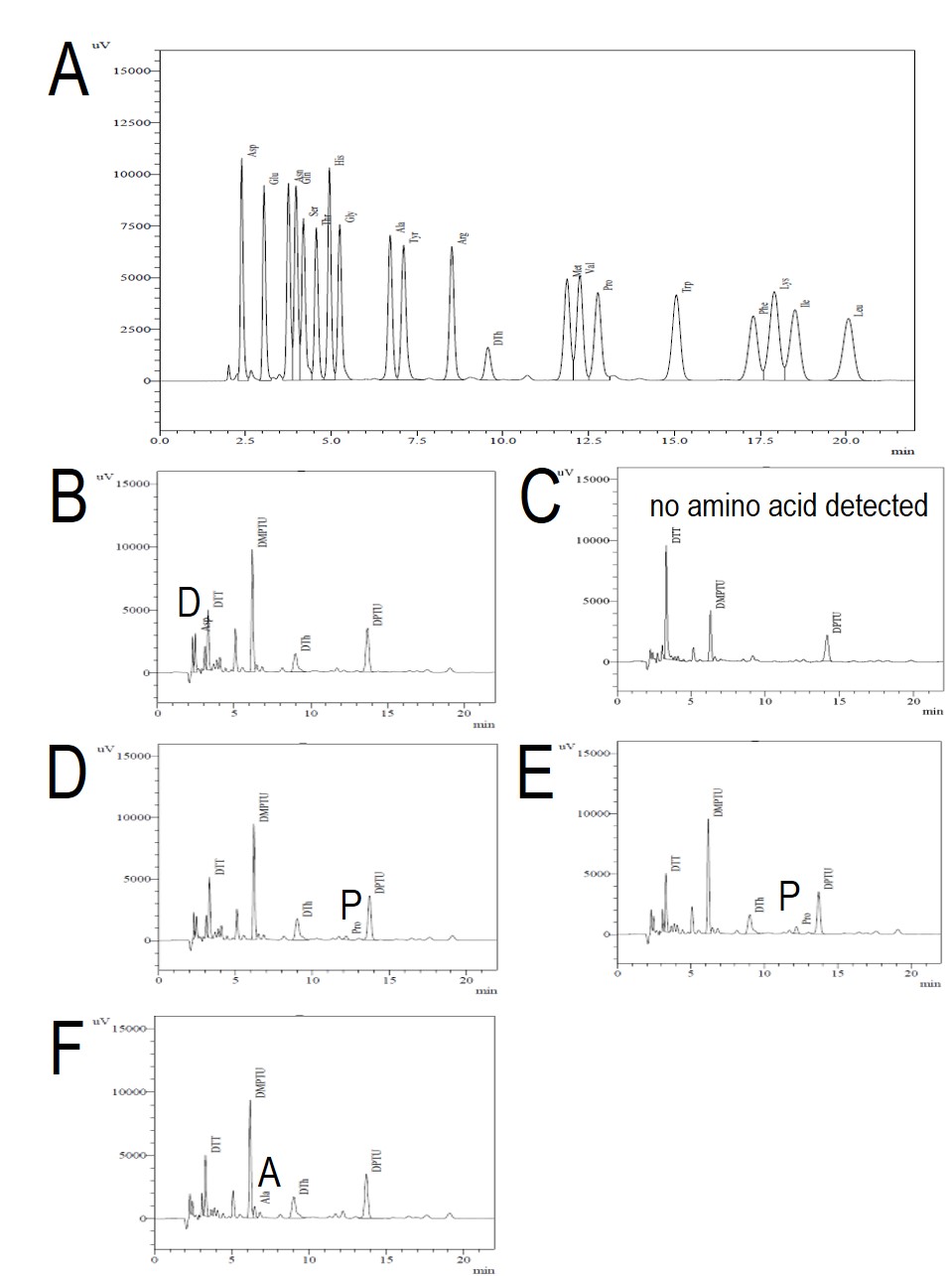


**Fig S2.** N-terminal sequencing identified cleavage-sites of Vip3Ad processed by *Spodoptera exigua* midgut juice. (A) Spectrum of 19 PTH-amino acids standards; (B)-(F) N-terminal [amino](javascript:void(0);) [acid](javascript:void(0);) identification of Vip3Aa activated-toxin.

**Fig S3**


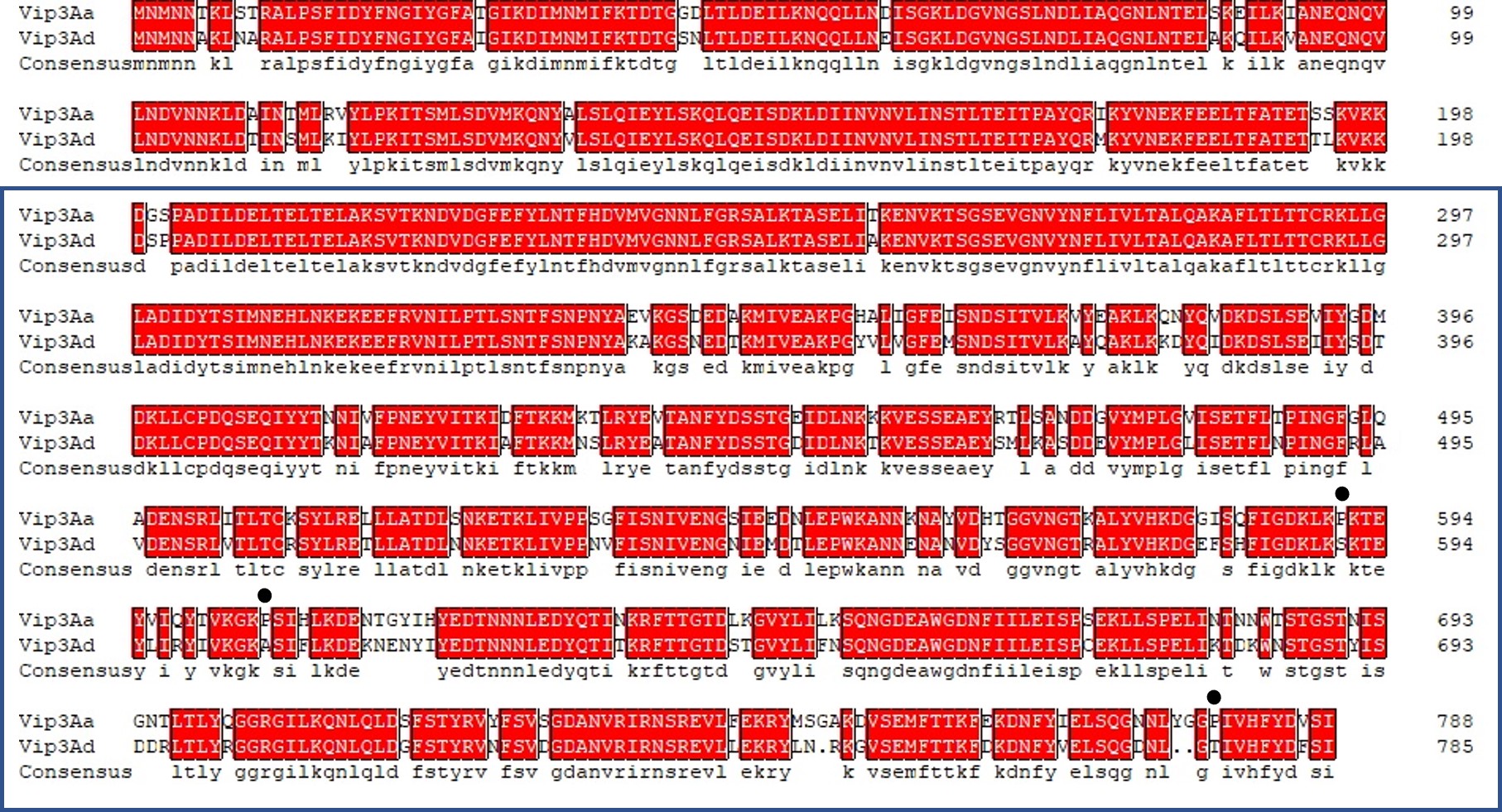


**Fig S3.** Amino acid sequence alignment of Vip3Aa and Vip3Ad. The sequences in blue box represented the 65 kDa activated-toxins of Vip3Aa and Vip3Ad. The positions of three extra prolines (P591, P605 and P779) was indicated as (●).
